# Hypotonic fluid reduce serum sodium compared to isotonic fluids during anesthesia induction in pediatric patients undergoing maxillofacial surgery-type of infusion affects blood electrolytes and glucose: an observational study

**DOI:** 10.1186/s12887-016-0650-6

**Published:** 2016-07-27

**Authors:** Kentaro Ouchi, Kazuna Sugiyama

**Affiliations:** Department of Dental Anesthesiology, Field of Maxillofacial Diagnostic and Surgical Sciences, Faculty of Dental Science, Kyushu University Graduate School, 3-1-1 Maidashi, Higashi-ku, Fukuoka, 812-8582 Japan

**Keywords:** Isotonic solutions, Hypotonic solutions, Hyponatremia, Hyperglycemia, General Anesthesia

## Abstract

**Background:**

Reportedly, administration of hypotonic fluids containing 30.8–74 mEq/L sodium with 5 % glucose may lead to serious hyponatremia or hyperglycemia. In Japan, hypotonic fluids containing 90 mEq/L sodium with 2.6 % glucose are commonly used. We compared blood electrolyte balance and blood glucose concentration with the use of isotonic (140 mEq/L sodium with 1 % glucose) versus hypotonic fluids in pediatric patients.

**Methods:**

We studied 77 children aged 5 months to 2 years who underwent oro-maxillofacial surgery and dental treatment under general anesthesia. Patients were categorized according to the fluids infused (hypotonic or isotonic). Blood samples were obtained from the dorsalis pedis artery between the conclusion of anesthesia induction and commencement of surgery. We compared blood sodium, potassium and glucose concentrations in the two fluid groups during the pre-anesthesia and post-anesthesia-induction periods.

**Results:**

There were no significant differences in pre-anesthesia values between isotonic (*n* = 35) and hypotonic groups (*n* = 42). There were significant differences between isotonic and hypotonic groups in post-anesthesia-induction concentrations of sodium (isotonic, 138.7 ± 1.4 mEq/L; hypotonic, 137.5 ± 1.3 mEq/L; *p* = 0.0003) and glucose (isotonic, 88.0 ± 9.4 mg/dL; hypotonic, 109.9 ± 18.4 mg/dL; *p* < 0.0001), while potassium concentrations were not significantly different (isotonic, 4.0 ± 0.3 mEq/L; hypotonic, 4.0 ± 0.2 mEq/L; *p* = 0.6615) between the two groups.

**Conclusion:**

Isotonic solution administration enables avoidance of serum sodium reduction and serum glucose elevation in infants, and may therefore enhance patient safety in comparison with hypotonic solutions.

**Trial registration:**

University Hospital Medical Information Network Clinical Trials Registry (UMIN000014648), registration 25 July 2014.

## Background

Intraoperative fluid administration in children is based on Holliday and Segar’s recommendations regarding the estimated metabolic requirements of patients on bed rest, which advocate the use of hypotonic fluids containing 30 mEq/L sodium with 5 % glucose [1–3]. In Japan, based on these recommendations, hypotonic fluids containing 90 mEq/L sodium with 2.6 % glucose are commonly used; these hypotonic fluids have higher concentrations of sodium and glucose than the recommendations [3]. In recent years, many studies and case reports have shown that hypotonic fluids containing 30.8–74 mEq/L sodium with 5 % glucose may lead to serious hyponatremia or hyperglycemia, and may occasionally result in permanent neurologic damage or death [2, 4–8]. As a result, some anesthesiologists prefer to administer isotonic fluids, especially during the perioperative period. However, even recently, hypotonic fluids are commonly administered intraoperatively [2, 3]. Under certain conditions, such as tracheal intubation and surgical procedures, vasopressin secretion increases, resulting in decreases in serum sodium [9]. Thus, sodium concentrations may decrease even during the short time period of anesthesia induction.

The aim of our observational study was to compare serum values of sodium, potassium and glucose at the conclusion of anesthesia induction, based on our routine blood tests at this time, after infusion of isotonic fluids containing 140 mEq/L sodium with 1 % glucose versus that after infusion of hypotonic fluids containing 90 mEq/L sodium with 2.6 % glucose. Thus, we measured blood electrolyte and glucose concentrations pre-anesthesia and post-anesthesia-induction.

## Methods

### Subjects

We studied American Society of Anesthesiologists (ASA) physical status I children aged 5 months to 2 years who underwent oro-maxillofacial surgery and dental treatment under general anesthesia at the Clinical Department of Dental Anesthesia, Kagoshima University Medical Dental Hospital from April 2012 to December 2014. Before entering the operating room, blood samples were obtained for biochemical and hematologic analysis for determination of blood electrolyte and glucose concentrations. This was defined as the pre-anesthesia value. Subjects with abnormal blood values of sodium (less than 136 and more than 145 mEq/L) and potassium (less than 3.5 and more than 5.0 mEq/L) were excluded from the study. Informed consent for oral surgery and for participation in this study under general anesthesia was obtained by written and verbal from each patient’s parent or guardian. This observational study was conducted with the approval of the ethics committee of Kagoshima University Medical Dental Hospital. The study was conducted in accordance with the Declaration of Helsinki. This study has adhered to the Strengthening the Reporting of Observational Studies in Epidemiology (STROBE) guidelines.

The necessary number of patients required for the study was determined based on a past study on serum sodium, in which the control serum sodium (± standard deviation) was 137 ± 1.7, according to which at least 31 patients in each group were considered necessary to achieve an approximately 1.7 % decrease in serum sodium by hypotonic infusion with an α risk of 0.05 and (1-β) of 0.95 [10].

### Anesthesia protocol

According to current recommendations, patients were allowed milk for up to 4.5 h and clear fluids for up to 2.5 h before surgery. After entering the operating room, anesthesia induction was commenced with administration of oxygen. General anesthesia was induced and maintained with inhalational administration of sevoflurane. An intravenous line for administration of the fluid was secured after loss of consciousness by slow induction, and fluid administration was commenced. Ventilation was performed to maintain EtCO_2_ between 35–40 mmHg, as assessed by capnography. The volume of infusion required to compensate for the dehydration arising from starvation for 3 h was calculated based on the 4-2-1 rule (4 ml for the first 10 kg body weight, 2 ml from 11 to 20 kg and 1 ml/kg/h for every kg above 20 kg), and was administered using an infusion pump. Between conclusion of anesthesia induction and commencement of surgery, blood samples were obtained from the dorsalis pedis artery for biochemical and hematologic analysis, for determination of blood electrolyte and glucose concentrations. This analysis is a standard part of our anesthesia routine. This value was defined as the post-anesthesia-induction value.

### Measurement of parameters

We measured blood sodium (mEq/L), potassium (mEq/L) and glucose (mg/dL) concentrations and the time interval between the start of infusion and blood examination. Blood analyses were performed using an ABL735 analyzer (Radiometer, Copenhagen, Denmark). Patients were categorized according to the fluids infused (isotonic or hypotonic); the composition of the fluids administered is shown in Table 1. The study authors did not intervene at to select the fluid. The anesthesiologist in charge of the case selected the fluid to be infused. Consequently, anesthesiologists A and B regularly gave the isotonic solution, and anesthesiologists C and D administered the hypotonic solution. Table 2 shows the patients’ demographic data. Seventy patients were categorized into the isotonic solution (*n* = 35) or hypotonic solution (*n* = 42) groups. Then, we compared blood sodium, potassium and glucose concentrations in the pre-anesthesia and post-anesthesia-induction periods and between the two groups.

**Table 1:** Composition of the study fluids

	Isotonic solution	Hypotonic solution
Sodium (mEq/L)	140	90
Chloride (mEq/L)	115	70
Potassium (mEq/L)	4	0
Glucose (g/L)	10	26
Calcium (mEq/L)	3	–
Magnesium (mEq/L)	2	–
Lactate (mEq/L)	–	20
Acetate (mEq/L)	25	–
Gluconate (mEq/L)	3	–
Citrate (mEq/L)	6	–
Manufacturers	Otsuka Pharmaceutical Factory, Inc., JAPAN	Terumo co., JAPAN
Trade names	Physio 140®	SOLDEM 1®

**Table 2:** Patient demographics

		Isotonic solution group (*n* = 35)	Hypotonic solution group (*n* = 42)	*p* value
Age (months)		16.4 ± 4.9	14.9 ± 6.0	0.2436 (NS)
Height (cm)		77.5 ± 6.6	74.7 ± 7.1	0.0781 (NS)
Weight (kg)		9.8 ± 1.4	9.3 ± 1.8	0.1897 (NS)
Male/Female		21/14	26/16	0.8645 (NS)
Medical conditions (number)	Asthma	3	5	
	Congenital heart disease	2	0	

There were no co-morbid renal conditions, which may have impacted sodium, potassium, glucose and ADH secretion, in either group. There were four patients on bronchodilator therapy (leukotriene inhibitor or inhalational steroid) among eight asthma patients

### Statistical analyses

Continuous demographic and laboratory variables were compared using the unpaired *t*-test, and the chi-square test was used for categorical variables. JMP software was used for statistical analysis, and *p* < 0.05 was regarded as being statistically significant. The results are presented as mean ± SD.

## Results

The time interval between the start of infusion and blood examination was not significantly different between the two groups (isotonic group, 48.7 ± 18.2 min; hypotonic group, 43.3 ± 9.5 min; *p* = 0.0990; mean, 45.1 ± 14.3 min). According to calculations, patients in both groups had received 5.5 ± 1.7 ml/kg of the infusate by the time of blood collection for measurement of the post-anesthesia-induction value.

There were no significant differences between isotonic and hypotonic groups in pre-anesthesia sodium (isotonic, 138.9 ± 1.5 mEq/L; hypotonic, 139.0 ± 1.5 mEq/L; *p* = 0.7331) and potassium concentrations (isotonic, 4.4 ± 0.3 mEq/L; hypotonic, 4.5 ± 0.3 mEq/L; *p* = 0.2084) (Table 3).

**Table 3:** Pre-anesthesia and post-anesthesia-induction blood concentrations of the measured biochemical parameters

	Pre-anesthesia		Post-anesthesia-induction			The change from Pre-anesthesia to post-anesthesia-induction	
	Sodium (mEq/L)	Potassium (mEq/L)	Sodium (mEq/L)	Potassium (mEq/L)	Glucose (mg/dL)	Sodium (mEq/L)	Potassium (mEq/L)
Isotonic solution group	138.9 ± 1.5	4.4 ± 0.3	138.7 ± 1.4	4.0 ± 0.3^a^	88.0 ± 9.4	−0.19 ± 1.99	−0.41 ± 0.41
Hypotonic solution group	139.0 ± 1.5	4.5 ± 0.3	137.5 ± 1.3^a^	4.0 ± 0.2^a^	109.9 ± 18.4	−1.49 ± 2.06	−0.52 ± 0.41
*p* value	0.7331 (NS)	0.2084 (NS)	0.0003	0.6615 (NS)	<0.0001	0.0064	0.2342 (NS)

^a^Comparison of pre-anesthesia and post-anesthesia-induction values indicated significant difference in potassium concentrations with the isotonic solution (*p* < 0.0001), sodium concentrations with hypotonic solution (*p* < 0.0001), and potassium concentrations with hypotonic solution (*p* < 0.0001)

In terms of the post-anesthesia-induction values, there were significant differences between isotonic and hypotonic solution groups in sodium (isotonic, 138.7 ± 1.4 mEq/L; hypotonic, 137.5 ± 1.3 mEq/L; *p* = 0.0003) and glucose concentrations (isotonic, 88.0 ± 9.4 mg/dL; hypotonic, 109.9 ± 18.4 mg/dL; *p* < 0.0001), while potassium concentrations were not significantly different (isotonic, 4.0 ± 0.3 mEq/L; hypotonic, 4.0 ± 0.2 mEq/L; *p* = 0.6615) (Table 3).

In comparisons between pre-anesthesia and post-anesthesia-induction values, the isotonic group indicated no significant difference in sodium concentrations (*p* = 0.5850), while post-anesthesia-induction potassium concentrations were significantly lower than pre-anesthesia values (*p* < 0.0001). In the hypotonic group, on the other hand, post-anesthesia-induction sodium and potassium concentrations significantly decreased as compared to their pre-anesthesia values (*p* < 0.0001 and *p* < 0.0001, respectively).

In terms of the change from pre-anesthesia to post-anesthesia-induction periods (post-anesthesia-induction value – pre-anesthesia value), there were significant differences between isotonic and hypotonic solution groups in the change in sodium concentrations (isotonic, −0.19 ± 1.99 mEq/L; hypotonic, −1.49 ± 2.06 mEq/L; *p* = 0.0064), while the change in potassium concentration was not significantly different (isotonic, −0.41 ± 0.41 mEq/L; hypotonic, −0.52 ± 0.41 mEq/L; *p* = 0.2342) (Table 3).

One patient who received the hypotonic solution developed hyponatremia (sodium less than 136 mEq/L), while none of the patients who received the isotonic solution developed hyponatremia This patient was a 1-year-6-month-old male, 80.0 cm tall, weighing 9.8 kg, with no relevant past history and who was undergoing palatoplasty. His pre-anesthesia sodium concentration was 139 mEq/L, post-anesthesia-induction sodium concentration was 134 mEq/L, and time interval to blood examination was 44 min. This patient had no symptoms/complications due to the hyponatremia, and it was not treated. Further, there was no associated hypoglycemia (glucose less than 70 mg/dL). On the other hand, four patients developed hyperglycemia (glucose more than 140 mg/dL) (Table 4).

**Table 4:** Demographics of patients who had hyperglycemia

Case	Age (months)	Height (cm)	Weight (kg)	Sex	Post-anesthesia-induction glucose (mg/dL)	Type of fluids
1	6	64.5	6.3	Female	143	Hypotonic
2	6	63.4	6.21	Male	148	Hypotonic
3	6	68.0	7.0	Female	142	Hypotonic
4	5	65.5	7.7	Female	146	Hypotonic

## Discussion

The main finding of this observational study was that post-anesthesia-induction infusion of a hypotonic solution containing 90 mEq/L sodium and 2.6 % glucose is associated with a decrease in serum sodium levels, while infusion of an isotonic solution containing 140 mEq/L sodium with 1 % glucose enables maintenance of serum sodium levels. Many reports have shown that hypotonic fluids, such as those containing 30.8 –74 mEq/L sodium, decrease blood sodium concentrations [8, 11]; in this study, administration of even a 90 mEq/L sodium solution resulted in a decrease in blood sodium concentrations. Many studies and case reports have shown that hypotonic fluids may lead to serious hyponatremia or hyperglycemia. In these reports, time between start of infusion and blood examination/assessment was long. Contrary, in this study, the time between start of infusion and blood examination was short (approximately 42 min).

In the perioperative period, the risk of developing hyponatremia is increased because of stress-induced secretion of antidiuretic hormone [12, 13]. The hyponatremia is associated with considerable morbidity and mortality [14, 15], including cerebral edema. In pediatric patients, even a small decrease in sodium concentrations can lead to cerebral herniation, due to the limited room available in the rigid skull to accommodate the swollen brain [12]. In addition, the ability of the pediatric brain to adapt to hyponatremia is poorer than that of adults [16]. One previous report showed that hypotonic fluids caused hyponatremia after surgery in 9 of 31 patients [17]. Other reports have shown that hypotonic fluids cause hyponatremia, with the resultant fall in sodium concentrations leading to serious neurologic outcomes in 2 of 40 patients who received hypotonic fluids [4]. When patients were divided based on the fluid they received (isotonic or hypotonic), patients who received hypotonic fluids had significant hyponatremia as compared to those who received isotonic fluids [18]. In this previous report, a decrease in sodium from 142 to 128 mmol/L led to cardiac arrest, and postmortem examination revealed brain cell swelling [4]. Thus, a decrease in sodium concentrations must be avoided.

In the current study, one patient who received hypotonic solutions developed hyponatremia, defined as serum sodium levels of 136 mEq/L or lower [19]. This patient had no symptoms/complications due to the hyponatremia. In this patient, blood samples following infusion were obtained at 42 min after commencement of fluid administration. This patient was considered to have mild hyponatremia, because the normal prescribed range of sodium is more than or equal to 136 mEq/L and less than 145 mEq/L [19]. Continued administration of hypotonic fluids to such patients can lead to worsening of the hyponatremia. In contrast, none of the patients who received isotonic solutions developed hyponatremia.

This study showed that hypotonic fluids reduce serum sodium even when administered for the short time period required for anesthesia induction. Vasopressin secretion increases under certain conditions, such as tracheal intubation, resulting in a decrease in serum sodium concentrations [9]. In a previous study, when patients were divided based on an elevated arginine vasopressin level (less than 3.5 or more than 3.5), patients with high arginine vasopressin levels who received hypotonic fluids had a significant fall in serum sodium as compared to those with low arginine vasopressin levels [20]. Additionally, overinfusion is believed to be one of the causes of reduced serum sodium following fluid infusion [21]. In this study, the volume of fluid administered was as required to compensate for the 3 h fasting period, based on the 4-2-1 rule, and was administered using an infusion pump. Thus, patients in both groups had received approximately 5.1 ml/kg of the infusate by the time of blood collection for measurement of the post-anesthesia-induction value. Hence, even though the time between start of infusion and blood examination was short (approximately 42 min), sodium concentrations decreased in the hypotonic group in this study. In contrast, sodium concentrations did not change in the isotonic group in this study, demonstrating that administration of isotonic fluids containing 140 mEq/l sodium enables maintenance of post-anesthesia-induction sodium concentrations, due to their similarity to normal blood sodium concentrations. It should be noted that although the difference between pre- and post-anesthesia-induction sodium concentrations in the hypotonic group was statistically significant, sodium concentrations in this study were still within the normal range and probably clinically not harmful [19].

Another observation of this study was that blood potassium concentrations were low irrespective of whether the administered fluid was a hypotonic solution that did not contain potassium or whether it was an isotonic solution containing 4 mEq/L of potassium. The invasiveness of anesthesia and surgery has been shown to decrease potassium concentrations [22], and adrenaline is known to affect serum potassium concentrations [23, 24]. Reportedly, patients with elevated serum adrenaline concentrations secondary to infringement stimulation have lower serum potassium concentrations [25]. This is probably due to adrenaline-induced intracellular movement of potassium, which is mediated via the beta 2 receptors of cells by way of membrane-bound Na/K ATPase [23]. The stimulus of endotracheal intubation also causes an increase in serum adrenaline concentrations [26]. Consequently, potassium concentrations decrease even fluids containing 4 mEq/L potassium, which is the normal concentration of potassium in the extracellular fluid. Changes in TCO_2_ are also important in potassium homeostasis. In this study, EtCO_2_ was maintained between 35 –40 mmHg, which would have helped maintain TCO_2_ at an approximately constant level. Besides, the isotonic fluid administered was a balanced electrolyte solution. Administration of balanced solutions reportedly prevents acidosis better than that achieved by hypotonic fluids.

In pediatric patients, surgical stress alone does not increase blood glucose concentrations when non-glucose infusions are used [27]. As 5 % glucose solutions are associated with an unacceptably high blood glucose concentration, solutions with a lower glucose concentration have been assessed in pediatric anesthesia [7]. Glucose solutions with a concentration of 2 %, which is a lower concentration than the 2.6 % glucose in the hypotonic solution in this study, are associated with an unacceptably high blood glucose concentration [7, 27]. Accordingly, solutions with an even lower glucose concentration have been assessed in pediatric anesthesia. It is well known that a severe lack of glucose enhances lipolysis, leading to ketogenesis. Use of 1 % glucose fluids reportedly maintains plasma glucose concentrations within the physiological range and prevents the development of ketoacidosis and metabolic acidosis, according to a study that assessed plasma pH but not ketone bodies [28]. Our results indicated that none of the patients in this study had post-anesthesia-induction blood glucose values suggestive of hypoglycemia.

Many studies have demonstrated the superiority of isotonic vs. hypotonic fluids, and several meta-analyses have been published on this topic [2, 4–8]. However, to date, hypotonic fluids are commonly administered intraoperatively [2]. In Japan as well, intraoperative hypotonic fluid administration is considered the norm. [3] Thus, this study serves as a quality improvement study and/or safety comparison of the use of hypotonic vs. isotonic fluids in pediatric patients.

Our study has several limitations. As a rule, in our facility, blood samples are obtained for biochemical and hematologic analysis between the conclusion of anesthesia induction and commencement of surgery. Further, blood samples following infusion were obtained at only one time point after anesthesia induction. More accurate assessment of the effect of infusion on pediatric blood electrolyte and glucose concentrations would be achieved by acquiring more frequent blood samples during the post-anesthesia-induction period. Yet, this study shows that in pediatric patients, hypotonic solutions are not suitable for administration for even the short time period required for anesthesia induction. Another limitation of our study is that vasopressin secretion was not assessed. Measurement of serum vasopressin concentrations may provide information about the mechanism of serum sodium reduction and hyponatremia. A further limitation of our study is the lack of randomization and blinding of the anesthesiologists. Yet, we believe that these limitations would not have significantly impacted our results, because it involved objective measurements of biochemical parameters in blood samples and this was an observational study. The next limitation of our study is that pre-anesthesia glucose levels were not compared. In Japan, because very few pediatric patients have abnormal blood glucose levels, their health insurance did not cover the measurement of glucose blood levels until around 2014, after the commencement of this study. Hence, there were no pre-anesthesia blood glucose values in some of our patients, preventing us from comparing them. We believe that this limitation would not have significantly impacted our results, because very few pediatric patients have abnormal blood glucose values. A further limitation of our study is that we did not examine plasma ketone bodies and nonesterified fatty acid concentrations. Evaluation of these biochemical parameters may have more effectively assessed the effects of the glucose concentrations of the different infusion fluids used. A further limitation of our study is that this study was an observational study that observed the results of routinely performed measurements. This study was an observational study in which we observed the results of routine measurements. The study authors did not intervene at all. Hence, the measurements were obtained from anesthesia records. There may also have been a selection bias in terms of which group the patients were enrolled in. However, since we first collected all the patients’ data during the study period, and then only included those subjects who did not meet the exclusion criteria described in the Methods section, we believe that selection bias did not occur. However, selection bias would have been more accurately eliminated if we had performed this study as a randomized intervention study.

## Conclusions

Our study shows that administration of hypotonic fluids tends to reduce serum sodium concentrations in pediatric patients, even when administered for a short period, as during anesthesia induction; in contrast, isotonic fluids help to avoid a reduction in serum sodium and elevation in serum glucose in infants and, therefore, may enhance patient safety.
